# Rag Defects and Thymic Stroma: Lessons from Animal Models

**DOI:** 10.3389/fimmu.2014.00259

**Published:** 2014-06-02

**Authors:** Veronica Marrella, Pietro Luigi Poliani, Luigi Daniele Notarangelo, Fabio Grassi, Anna Villa

**Affiliations:** ^1^Milan Unit, Institute of Genetics and Biomedic Research, National Research Council, Milan, Italy; ^2^Istituto Clinico Humanitas, Istituto di Ricovero e Cura a Carattere Scientifico, Rozzano, Italy; ^3^Pathology Unit, Department of Molecular and Translational Medicine, University of Brescia, Brescia, Italy; ^4^Division of Immunology, Boston Children’s Hospital, Boston, MA, USA; ^5^Institute for Research in Biomedicine, Bellinzona, Switzerland

**Keywords:** thymus, Rag deficiency, Omenn and leaky SCID models, central tolerance, thymic reconstitution, thymic cross-talk

## Abstract

Thymocytes and thymic epithelial cells (TECs) cross-talk is essential to support T cell development and preserve thymic architecture and maturation of TECs and Foxp3^+^ natural regulatory T cells. Accordingly, disruption of thymic lymphostromal cross-talk may have major implications on the thymic mechanisms that govern T cell tolerance. Several genetic defects have been described in humans that affect early stages of T cell development [leading to severe combined immune deficiency (SCID)] or late stages in thymocyte maturation (resulting in combined immunodeficiency). Hypomorphic mutations in SCID-causing genes may allow for generation of a limited pool of T lymphocytes with a restricted repertoire. These conditions are often associated with infiltration of peripheral tissues by activated T cells and immune dysregulation, as best exemplified by Omenn syndrome (OS). In this review, we will discuss our recent findings on abnormalities of thymic microenvironment in OS with a special focus of defective maturation of TECs, altered distribution of thymic dendritic cells and impairment of deletional and non-deletional mechanisms of central tolerance. Here, taking advantage of mouse models of OS and atypical SCID, we will discuss how modifications in stromal compartment impact and shape lymphocyte differentiation, and vice versa how inefficient T cell signaling results in defective stromal maturation. These findings are instrumental to understand the extent to which novel therapeutic strategies should act on thymic stroma to achieve full immune reconstitution.

## Introduction

Thymocytes and thymic epithelial cells (TECs) cross-talk is essential to support T cell development and preserve thymic architecture and maturation of TECs and Foxp3^+^ natural regulatory T (nTreg) cells. In particular, deletion of self-reactive thymocytes in the thymic medulla is based on the recognition of self-antigens that are presented by medullary TECs (mTECs) and thymic dendritic cells (DCs). In this process, a key role is played by the autoimmune regulator (AIRE), a transcription factor expressed by a subset of mature mTEC that drives the expression of tissue-restricted antigens (TRAs), thus mediating negative selection of autoreactive thymocytes (1, 2). In addition, mature TECs from the Hassall corpuscles secrete thymic stromal lymphopoietin (TSLP), a cytokine that acts through thymic DCs and activates them to instruct self-reactive T cells to be diverted into Foxp3^+^ nTreg cells (3). These findings highlight the critical role played by the thymus not only in the generation of a diversified and functional T cell repertoire, but also in the prevention of autoimmune manifestations. To this end, defects in T cell development represent a valuable model for studying mechanisms by which severe impairment in thymopoiesis may impinge on thymic stromal cell homeostasis and deletional and non-deletional mechanisms (4). In particular, severe combined immune deficiency (SCID) includes a heterogeneous group of genetic disorders that abolish T cell development at early stage of T cell differentiation by affecting survival of lymphoid progenitors (as in adenosine deaminase deficiency and reticular dysgenesis), interleukin (IL)-mediated expansion of lymphoid progenitors (as in patients with mutations in the γ common chain, JAK3, or IL-7 receptor), V(D)J recombination [as in recombination activating gene (Rag)1 and 2, and Artemis deficiency] in lymphoid precursors and signaling through the pre-T cell receptor (mutations of CD3δ, CD3ε, CD3ζ, and CD45) (5). Null mutations in these SCID-causing genes are associated with a virtual lack of circulating T lymphocytes. However, hypomorphic mutations in the same genes may allow for development of a restricted number of T lymphocytes with limited repertoire diversity, which, when exported to the periphery, may infiltrate target tissues and cause autoimmunity and organ dysfunction, as in patients with Omenn syndrome (OS) (6–10). Finally, defects in T cell development that compromise thymocyte development beyond the CD4^+^ CD8^+^ [double-positive (DP)] stage result in combined immunodeficiency with residual number of circulating T lymphocytes that show abnormal phenotype and function. In particular, impaired production of single-positive (SP) CD4^+^ cells is observed in major histocompatibility complex (MHC) class II deficiency, whereas generation of SP CD8^+^ lymphocytes is compromised in ZAP70 deficiency (11, 12). Association of these conditions with immune dysregulation has been reported, although not as frequently as in OS due to hypomorphic mutations in SCID-causing genes (13).

In this review, we will focus the discussion on the contribution of thymic microenvironment on the pathogenesis of peripheral immune pathology in the presence of residual V(D)J recombination activity. To this end, we will discuss findings observed in *Rag2^R229Q/R229Q^* and *Rag1^S723C/S723C^* mutant mice, which represent a valuable model of OS and atypical SCID, respectively (14–18). Collectively, we provide evidence that abnormalities of thymic stroma secondary to impaired development of T lymphocytes may affect key mechanisms of immune tolerance and ultimately result in severe manifestations of immune dysregulation.

## Mouse Models of Leaky SCID and OS

**Figure 1:**
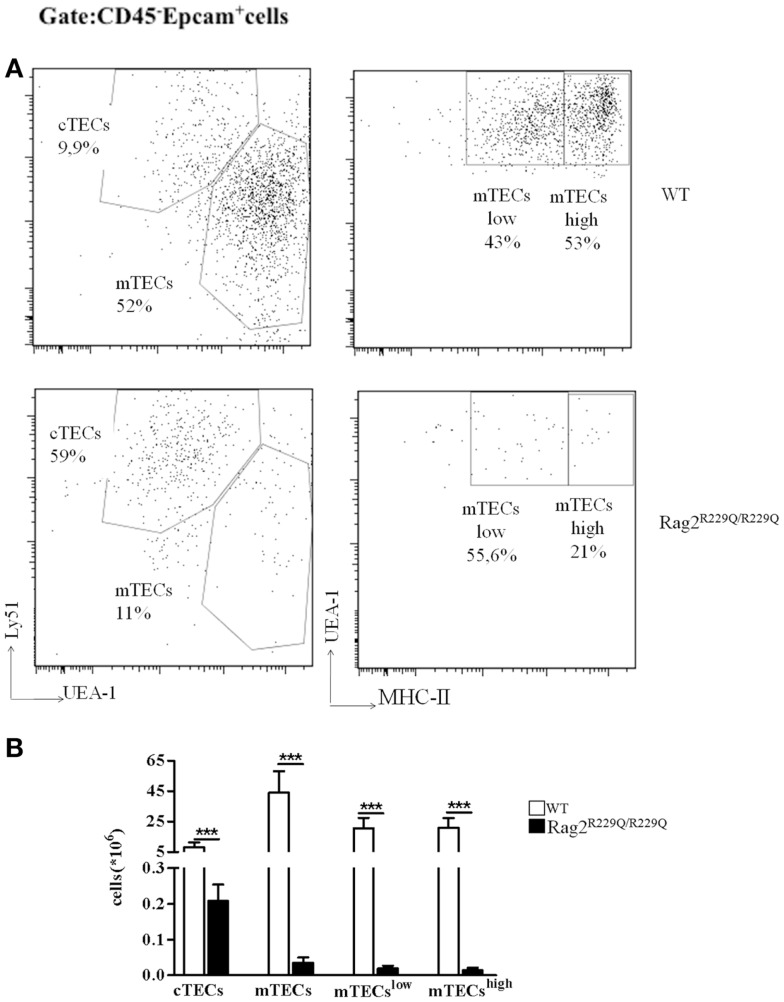
**Thymic epithelial cell alterations in *Rag2^R229Q/R229Q^* mouse**. **(A)** Representative FACS analysis of thymic epithelial compartment defined as Epcam^+^ cells in the CD45 negative fraction obtained after enzymatic digestion and enriched through AUTOMACS selection. Digested cells were stained with specific antibodies (Ly51, UEA-1, and MCH-II) to identify the different epithelial subsets as indicated in the dot-plots. Numbers represent the percentage within the indicated regions. **(B)** Graphic representation of absolute numbers for each epithelial population obtained upon enzymatic digestion in all mice analyzed (WT, *n* = 5; *Rag2^R229Q/R229Q^, n* = 9). Groups were analyzed with Prism software (GraphPad) using a two-tailed Mann–Whitney unpaired test. Data are presented as mean ± SD. *P*-values of <0.05 were considered significant.

Mutations of Rag genes result in a variety of clinical and immunological phenotypes. In particular, while null mutations cause a severe block in T and B cell development (T^−^ B^−^ SCID), hypomorphic *Rag1* and *Rag2* mutations may cause a spectrum of phenotypes, including OS, atypical SCID, combined immune deficiency with expansion of TCRαβ^+^ T cells, and combined immune deficiency with granuloma and/or autoimmunity (CID-G/A) despite their common molecular mechanisms underlying the disease (19–25). While all of these conditions associated with hypomorphic *Rag* mutations are characterized by residual development of T (and in some cases, B) lymphocytes, some of them (especially OS and CID-G/A) present with prominent immune dysregulation. However, the cellular and molecular mechanisms underlying autoimmunity have remained poorly defined until recently, when animal models of OS and leaky SCID have become available (16, 17, 26). In particular, Khiong and colleagues have reported on a spontaneously occurring mouse mutant (named MM) in which a homozygous point mutation in the *Rag1* gene (R972Q) was associated with a high proportion of memory T cells in the periphery. Although MM mice showed skin redness when shaved, no T cells infiltration was observed in the tissues and no obvious signs were reported, making this mutant strain a model of leaky SCID, in which T and B cells are present in low number and T cells are predominantly activated, but no obvious signs of autoimmunity are present (26). In another mouse model, homozygosity for the Rag1 S721C mutation was associated with impaired T cell development, presence of oligoclonal, activated T cells, profound B cell lymphopenia, and yet significant serum levels of immunoglobulin (15, 17, 18). Although only a minority of *Rag1^S723C/S723C^* mice developed signs of OS, T cell infiltrates in peripheral tissues, and autoantibodies to double stranded DNA (dsDNA) and other self-antigens were demonstrated in a significant proportion of mutant mice 15. Immune dysregulation was even more prominent in another mutant mouse model carrying a homozygous *Rag2* R229Q mutation, as shown by expansion of oligoclonal activated T cells infiltrating target organs including skin, gut, liver, and lung and by the presence of high IgE serum levels and autoantibodies, despite the absence of circulating B cells (14, 16). Of note, immune dysregulation in *Rag1^S723C/S723C^* and *Rag2^R229Q/R229Q^* mutants was associated with profound thymic abnormalities, with lack of corticomedullary demarcation (CMD), and impaired maturation of TECs (17, 27). In particular, both *Rag1^S723C/S723C^* and *Rag2^R229Q/R229Q^* mice displayed altered maturation of mTECs, as indicated by the virtual absence of expression of claudin-4 (Cld4) and Ulex europaeus Agglutinin 1 (UEA-1) ligand. Furthermore, analysis of cytokeratin (CK) expression in the thymus revealed abundance of CK8^+^ CK5^+^ cells, which represent immature TEC progenitors and a severe reduction of CK8^−^ CK5^+^ mTECs. FACS analysis labeling CD45^−^ Epcam^+^ thymic stromal cells with UEA-1 and Ly51 specific antibodies for mTECs and cTECs, respectively, have demonstrated the increased frequency of cTECs with consequent reduction in mTEC compartment in *Rag2^R229Q/R229Q^* mouse compared to WT (Figure 1A). However, all epithelial populations were significantly diminished in number given the dramatic reduction in total thymic cellularity (Figure 1B). Defective maturation of mTECs in *Rag1^S723C/S723C^* and *Rag2^R229Q/R229Q^* mice was associated with severe reduction of AIRE-expressing cells and markedly reduced expression of TRAs, such as cytochrome P450, insulin 2, glutamic acid decarboxylase 67, and fatty acid-binding proteins (17, 27). These defects inevitably lead to a severe impairment in the process of negative selection of autoreactive T cells clones.

Unexpectedly, abnormalities of thymic DCs, which are involved in promoting negative selection of self-reactive thymocytes and in the generation of nTregs, were also demonstrated in both mutant models. In particular, a relative abundance of CD11c^int^ CD45RA^+^ plasmacytoid DCs (pDCs), and a decreased proportion of CD11c^+^ CD45RA^−^ conventional DCs (cDCs) was demonstrated in *Rag1^S723C/S723C^* mice (17). A severe reduction of both cDCs and pDCs was demonstrated in *Rag2^R229Q/R229Q^* mice, and was associated with a random distribution of these DC subsets throughout the thymus. Furthermore, a significant reduction in the expression of MHC-II and CD86 was found in both DC subset populations, suggesting impairment in DC maturation process (28). Of note, impaired maturation of mTECs, defective expression of AIRE and reduced number of thymic DCs have been also reported in patients with hypomorphic mutations of genes involved in early stages of T cell development (29, 30). While the mechanisms accounting for thymic DC abnormalities in mice and patients with hypomorphic *Rag* mutations remain poorly defined, they have important consequences on maintenance of immune homeostasis. In particular, cDCs have been described to contribute to the generation of nTregs (31). Consistent with this, a reduced number of Foxp3^+^ nTreg cells have been observed in both *Rag1^S723C/S723C^* and *Rag2^R229Q/R229Q^* mice, as well as in patients with Rag-dependent OS (16, 17, 30).

Altogether, the study of animal models carrying hypomorphic *Rag* mutations has demonstrated that defective T cell lymphopoiesis affects maturation and function of thymic stroma, and impinges on both deletional and non-deletional mechanisms of immune tolerance, thereby providing important insights on the pathophysiology of OS.

## Animal Studies to Target Thymic Stroma in Rag Deficiencies

### Gene therapy in *Rag1* knock-out mice

An additional demonstration of the importance of thymic lymphostromal cross-talk has been provided by recent data demonstrating that inefficient T cell reconstitution following gene therapy in *Rag*1 deficient mice results in an OS-like phenotype (32). In this particular model, the majority of *Rag1* knock-out (KO) mice treated with lentiviral vectors carrying codon optimized human Rag1 cDNA driven by ubiquitous and cell type-restricted promoters showed low level of T cell reconstitution. In this setting of T cell lymphopenia, homeostatic proliferation led to peripheral T cell expansion, associated with a restricted T cell repertoire and a tendency of T cells to infiltrate peripheral tissues such as skin, lung, and kidney. Impaired T cell reconstitution, with reduced thymic cellularity, led to only partial rescue of thymic stroma morphology and maturation, with focal areas of CMD and low number of mature mTECs expressing AIRE. By contrast, transplantation of wild-type bone marrow cells into *Rag1*^−/−^ mice leads to the rescue of thymopoiesis and thymic stroma architecture, with presence of a well-defined CMD and a normal distribution of cTEC, immature and mature TEC expressing UEA-1 ligand and AIRE. Of note, 2 months after treatment, 50% of gene therapy-treated mice started to develop skin rash and wasting syndrome, which in some cases led to death. Poor thymic reconstitution correlated with massive lymphocytic infiltrates in peripheral tissues and presence of activated (CD44^+^CD69^+^) T cells, despite the presence of Foxp3^+^ cells. Moreover, gene therapy-treated mice showed a significant increase in serum IgE levels, presence of anti-dsDNA antibodies and increased BAFF levels, which represent typical biomarkers of immune dysregulation in patients and animal models of OS (14, 18). These data indicate that inadequate rescue of Rag1 expression leads to poor reconstitution of T and B cells and is insufficient to restore thymic stroma architecture, maturation of AIRE and TSA-expressing mTECs, and induction of both T and B cell tolerance. In this scenario, development of a limited number of T and B lymphocytes and inability to maintain efficient tolerance checkpoints lead to the development of OS-like manifestations. Overall, these data illustrate the importance of T cell reconstitution for restoring the differentiation and maturation of TECs, and emphasize the relevance of thymic stroma in ensuring immune tolerance and preventing thymic egress of autoreactive T cell clones.

### Anti-CD3ε mAb treatment in Rag2^R229Q/R229Q^ mouse model of OS

**Figure 2:**
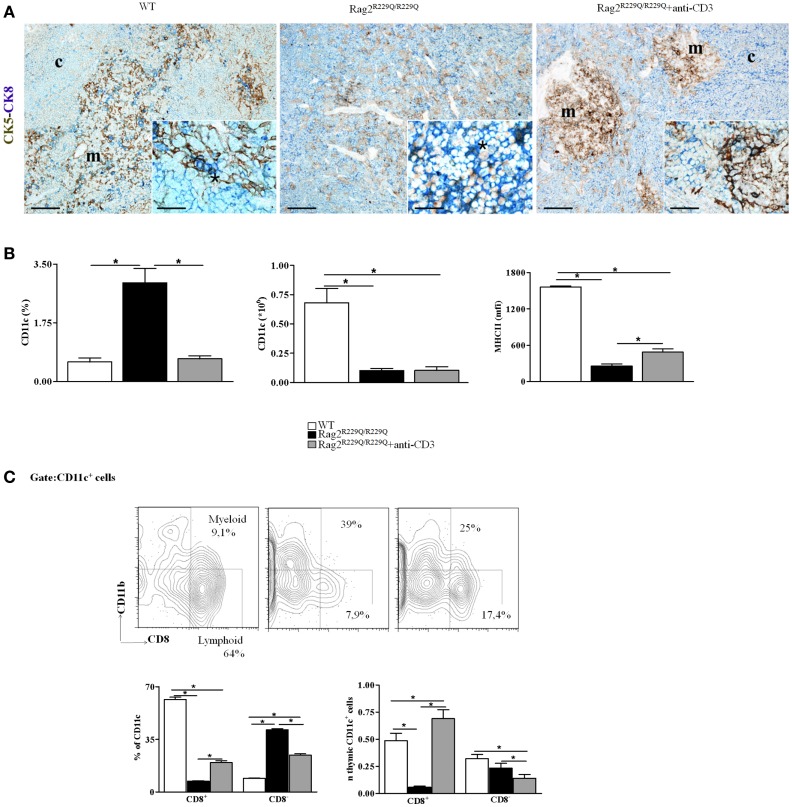
**Anti-CD3ε mAb administration enhances thymic epithelium compartmentalization and maturation and modifies thymic DCs frequency and distribution in *Rag2^R229Q/R229Q^* newborns**. **(A)** Left panel shows representative immunohistochemistry from WT thymus displaying a well-defined corticomedullary differentiation and normal compartmentalization of CK8^+^CK5^−^ cTECs (CK8, blue) and CK8^−^CK5^+^ mTECs (CK5, brown). mTECs show mature morphology with large cytoplasm and delicate CK5 positivity with rare double-positive CK8^+^CK5^+^ immature TECs disposed along the corticomedullary junction (asterisk within inset). Corticomedullary differentiation and maturation of TECs are profoundly impaired in *Rag2^R229Q^/^R229Q^* mouse (middle panel) in which immature TECs expressing both CK5 and CK8 are highly represented (asterisk within inset). Anti-CD3ε mAb administration enforces maturation of TECs leading to compartmentalization of CK8^+^CK5^−^ cTECs and CK8^−^CK5^+^ mTECs (right panel), although mTECs are still closely packed and irregularly distributed with intense CK5 positivity as compared to the normal medulla (detail of morphology within inset). Double immunohistochemical staining: CK5 (brown staining) and CK8 (blue staining). (m, medulla; c, cortex; scale bars corresponds to 200 and 50 μm for 10× and 40× (insets) original magnification, respectively). **(B)** Graphic representation of the percentage and absolute number of CD11c^+^ cells in the thymus of all mice analyzed (WT, *n* = 7; *Rag2^R229Q/R229Q^, n* = 7; *Rag2^R229Q/R229Q^* + anti-CD3 *n* = 9). The last graph on the right indicates mean fluorescence intensity (MFI) of MHC-II expression on total CD11c^+^ cells in all mice analyzed (WT, *n* = 5; *Rag2^R229Q/R229Q^, n* = 6; *Rag2^R229Q/R229Q^* + anti-CD3 *n* = 4). **(C)** Representative dot plot indicating the distribution of myeloid (CD8^−^) and lymphoid (CD8^+^) populations in the gate of CD11c^+^ cells (upper panel). Statistics of the percentage and the absolute numbers of CD8^+^ and CD8^−^ CD11c^+^ in all mice analyzed (WT, *n* = 5; *Rag2^R229Q/R229Q^, n* = 4; *Rag2^R229Q/R229Q^* + anti-CD3 *n* = 4) (lower panel). Groups were analyzed with Prism software (GraphPad) using a two-tailed Mann–Whitney unpaired test. Data are presented as mean ± SD. *P*-values of <0.05 were considered significant.

As previously described, OS is an atypical SCID in which the coexistence of immunodeficiency and autoimmunity remains an intriguing aspect that needs to be further investigated. Thanks to availability of the *Rag2^R229Q/R229Q^* mouse model, we have studied various mechanisms that contribute to the pathogenesis of autoimmune manifestations of OS. We have demonstrated that in addition to hypomorphic *Rag* defect leading to generation of a limited number of T cells, severe defects in thymic epithelial compartment occur, which contribute to the escape of autoreactive T cells that invade the periphery triggering autoimmunity (24). This model represents also a valuable tool to evaluate the effects of TCR signaling on maturation of the thymic stromal compartment. To this end, we evaluated the *in vivo* effect of anti-CD3ε monoclonal antibody (mAb) administration in neonatal and adult mice. While no significant changes were noticed in the thymus of adult treated mice, injection of anti-CD3ε mAb at neonatal age resulted in a dramatic amelioration of the epithelial compartment and peripheral immunopathology. In particular, treatment was associated with a marked reduction in the frequency of effector/memory T cells in the periphery and a significant decrease in interferon-γ (IFN-γ) and tumor necrosis factor-α (TNF-α) production by peripheral T cells. These changes were paralleled by significant modification in thymus morphology, with appearance of distinct areas of CMD and significant improvement of the medullary/cortical ratio (27). Double staining for CK5 and CK8 further confirmed these findings by revealing the presence of well-defined cortical and medullary areas showing that anti-CD3ε mAb treatment enforces maturation of TECs leading to compartmentalization of CK8^+^CK5^−^ cTECs and CK8^−^CK5^+^ mTECs (Figure 2A). Moreover, we have described an increase in the presence of UEA-1^+^ cells, although the formation of UEA-1^+^ mature mTECs clusters was not fully restored.

Furthermore, treatment with anti-CD3ε mAb normalized the frequency while did not change the absolute number of total thymic DCs and significantly increased MHC-II expression in this population normally down-regulated in *Rag2^R229Q/R229Q^* mice respect to WT counterpart (Figure 2B). More interestingly, anti-CD3ε mAb treatment induced a redistribution of the two thymic DCs main subsets CD8^−^ (myeloid) and CD8^+^ (lymphoid) (Figure 2C). The improvement of thymic stroma architecture and maturation were associated with a reduction in tissue infiltrates, as demonstrated by the reduced frequency of CD4^+^ and CD8^+^ cells in the skin, gut, lung, and liver. Altogether, these data indicate that treatment with anti-CD3ε mAb has a beneficial effect on thymic stroma and on peripheral immunopathology, and may pave the way for similar therapeutic modalities aiming at improving immune function and reducing signs of immune dysregulation in patients with SCID characterized by poor thymic maturation, while waiting for definitive treatment based on hematopoietic cell transplantation.

## Conclusion

Thymocytes and TEC cross-talk are fundamental for the maintenance of thymic architecture and function. Investigation on thymic morphology and immunophenotype in SCID patients and in parallel analysis of murine models of OS and Leaky SCID have revealed the extent to which altered thymic cross-talk might lead to immune dysregulation and ultimately cause peripheral immunopathology. Thymic stromal improvement and amelioration of peripheral immunopathology upon anti-CD3ε mAb administration in OS mouse model have further highlighted the contribution of thymic stroma in the pathogenesis of immune dysregulation. In parallel, poor immunological reconstitution observed in the preclinical study of gene therapy caused by inadequate Rag1 expression has further emphasized the relevance of stromal thymic compartment in the induction and maintenance of immune tolerance. Overall these findings further define the role of thymic epithelium in immune reconstitution and indicate that cTECs and mTECs full restoration has to be achieved to prevent immune dysregulation.

## Conflict of Interest Statement

The authors declare that the research was conducted in the absence of any commercial or financial relationships that could be construed as a potential conflict of interest.
