# Corrigendum: Rag Defects and Thymic Stroma: Lessons from Animal Models

**DOI:** 10.3389/fimmu.2014.00407

**Published:** 2014-08-25

**Authors:** Veronica Marrella, Pietro Luigi Poliani, Luigi Daniele Notarangelo, Fabio Grassi, Anna Villa

**Affiliations:** ^1^Milan Unit, Institute of Genetics and Biomedic Research, National Research Council, Milan, Italy; ^2^Istituto Clinico Humanitas, Istituto di Ricovero e Cura a Carattere Scientifico, Rozzano, Italy; ^3^Pathology Unit, Department of Molecular and Translational Medicine, University of Brescia, Brescia, Italy; ^4^Division of Immunology, Boston Children’s Hospital, Boston, MA, USA; ^5^Institute for Research in Biomedicine, Bellinzona, Switzerland

**Keywords:** hymus, rag deficiency, omenn and leaky SCID models, central tolerance, thymic reconstitution, thymic crosstalk

Author list for the article “Rag defects and thymic stroma: lessons from animal models” should be as follows:

Veronica Marrella^1,2^, Pietro Luigi Poliani^3^, Luigi Daniele Notarangelo^4^, Fabio Grassi^5^, and Anna Villa^1,2^*

^1^Milan Unit, Institute of Genetics and Biomedic Research, National Research Council, Milan, Italy

^2^Istituto Clinico Humanitas, Istituto di Ricovero e Cura a Carattere Scientifico, Rozzano, Italy

^3^Pathology Unit, Department of Molecular and Translational Medicine, University of Brescia, Brescia, Italy

^4^Division of Immunology, Boston Children’s Hospital, Boston, MA, USA

^5^Institute for Research in Biomedicine, Bellinzona, Switzerland

The original article has been updated.

## Conflict of Interest Statement

The authors declare that the research was conducted in the absence of any commercial or financial relationships that could be construed as a potential conflict of interest.

